# Potential Benefits of Epidermal Growth Factor for Inhibiting Muscle Degrative Markers in Rats with Alcoholic Liver Damage

**DOI:** 10.3390/ijms24108845

**Published:** 2023-05-16

**Authors:** Qian Xiao, Yi-Hsiu Chen, Ya-Ling Chen, Yu-Shan Chien, Li-Hsuan Hsieh, Hitoshi Shirakawa, Suh-Ching Yang

**Affiliations:** 1 School of Nutrition and Health Sciences, Taipei Medical University, Taipei 11031, Taiwan; 2 Laboratory of Nutrition, Graduate School of Agricultural Science, Tohoku University, Sendai 980-8857, Japan; 3 Research Center of Geriatric Nutrition, College of Nutrition, Taipei Medical University, Taipei 11031, Taiwan; 4 Nutrition Research Center, Taipei Medical University Hospital, Taipei 11031, Taiwan; 5 School of Gerontology and Long-Term Care, College of Nursing, Taipei Medical University, Taipei 11031, Taiwan

**Keywords:** ethanol, epidermal growth factor, alcoholic liver injury, microbiota, rat

## Abstract

This study investigated the beneficial effects of epidermal growth factor (EGF) on muscle loss in rats with chronic ethanol feeding. Six-week-old male Wistar rats were fed either a control liquid diet without EGF (C group, *n* = 12) or EGF (EGF-C group, *n* = 18) for two weeks. From the 3rd to 8th week, the C group was divided into two groups. One was continually fed with a control liquid diet (C group), and the other one was fed with an ethanol-containing liquid diet (E group); moreover, the EGF-C group was divided into three groups, such as the AEGF-C (continually fed with the same diet), PEGF-E (fed with the ethanol-containing liquid diet without EGF), and AEGF-E (fed with the ethanol-containing liquid diet with EGF). As a result, the E group had significantly higher plasma ALT and AST, endotoxin, ammonia, and interleukin 1b (IL-1b) levels, along with liver injuries, such as hepatic fatty changes and inflammatory cell infiltration. However, plasma endotoxin and IL-1b levels were significantly decreased in the PEGF-E and AEGF-E groups. In addition, the protein level of muscular myostatin and the mRNA levels of forkhead box transcription factors (FOXO), muscle RING-finger protein-1 (MURF-1) and atorgin-1 was increased considerably in the E group but inhibited in the PEGF-E and AEGF-E groups. According to the principal coordinate analysis findings, the gut microbiota composition differed between the control and ethanol liquid diet groups. In conclusion, although there was no noticeable improvement in muscle loss, EGF supplementation inhibited muscular protein degradation in rats fed with an ethanol-containing liquid diet for six weeks. The mechanisms might be related to endotoxin translocation inhibition, microbiota composition alteration as well as the amelioration of liver injury. However, the reproducibility of the results must be confirmed in future studies.

## 1. Introduction

Alcoholic liver disease (ALD) is among the most severe outcomes of chronic liver disease [1]. The liver is the main organ that metabolizes alcohol. Hepatocytes oxidize ethanol into acetaldehyde, a highly reactive and toxic byproduct that may contribute to tissue damage, mainly through the alcohol dehydrogenase and cytochrome P450 2E1 (CYP2E1) systems [2]. Acetaldehyde is oxidized by aldehyde dehydrogenase in mitochondria to produce acetate, which then enters the tricarboxylic acid cycle [2]. CYP2E1 activation results in the excessive generation of reactive oxygen species, which causes oxidative stress and thus accelerates liver damage [2]. The presence of high levels of blood lipopolysaccharides (LPSs), a condition known as endotoxemia, leads to the activation of the hepatic Toll-like receptor 4 (TLR4)/myeloid differentiation factor 88 (Myd88) pathway after long-term alcohol consumption [3]. Proinflammatory cytokines stimulated by alcohol metabolites additionally or synergistically with LPS translocated from gut bacteria accelerate liver damage [3,4].

In addition to liver damage, muscle loss or sarcopenia is a common complication of systemic inflammatory disorder and malnutrition [5]. Disruption of muscle homeostasis can be attributed to the gut–muscle and liver–muscle axes. In the gut–muscle axis, alcohol-induced increases in paracellular permeability, leaky gut, and dysbiosis can cause endotoxemia [5]. Subsequently, anabolic resistance and mammalian target of rapamycin (mTOR)-dependent autophagy is induced in the muscles. In the liver–muscle axis, hyperammonemia induced by liver dysfunction causes an increase in the muscular myostatin level. Hyperammonemia transcriptionally activates the synthesis of myostatin in cirrhosis by triggering the translocation of the nuclear factor kappa-light-chain-enhancer of activated B cells [6]. Myostatin potentiates muscle RING-finger protein-1 (MURF-1) and atrogin-1 mRNA transcription through forkhead box transcription factors (FOXO), and Smad2/3 cascades [7]. In addition, myostatin can inhibit muscular protein synthesis by reducing the levels of p70S6K and 4EBP1, which mTOR phosphorylates to maintain homeostasis between muscular protein synthesis and degradation [7,8]. Given increased LPS levels and dysbiosis in ALD, maintaining gut health can prevent liver damage and muscle loss in patients with this disease [9,10].

Epidermal growth factor (EGF) comprises 53 amino acids with nonglycosylated and stable properties [11]. EGF is found in various body fluids and is secreted by Brunner’s glands; it is crucial for cell growth and proliferation [12,13]. EGF participates in cell signaling by binding to its receptors, which are widely distributed in the basolateral membrane of the gastrointestinal tract. By phosphorylating tyrosine kinase, EGF triggers cell proliferation and differentiation pathways, such as the mTOR and protein kinase B (Akt) pathways [14,15]. Moreover, EGF signaling maintains the normal epithelial barrier provided by intestinal stem cells [16]. Thus, EGF is considered a vital regulator of the gut barrier [13]. In addition, EGF protects epithelial permeability, which is maintained by tight junctions from the impairment inducers, such as LPS. In vitro, EGF has reportedly upregulated occludins, zonula occludens-1 (ZO-1), and claudin-3 in Caco-2 [17,18] and NRC-1 [19] cells treated with hydrogen peroxide or acetaldehyde. In ALD rats, EGF treatment reduced the hepatic inflammatory response and *Escherichia coli* cells in fecal flora [20].

Studies demonstrating the beneficial effects of EGF on alcohol-associated muscle damage with a focus on the gut–liver–muscle axis are limited. Therefore, this study investigated the effects of EGF supplementation before or during the ethanol feeding period in rats. Moreover, this study determined the relationship between muscular protein homeostasis and intestinal damage (such as that related to permeability and microbiota composition) under long-term ethanol ingestion in rats receiving EGF supplementation.

## 2. Results

### 2.1. Food Intake and Efficiency, Ethanol Consumption, and EGF Supplementation 

No difference in food intake was observed among the groups (Table 1). Moreover, the ethanol intake was similar among the E, PEGF-E, and AEGF-E groups. However, the food efficiency was significantly lower in the ethanol-fed groups than in the control diet–fed groups. For example, the EGF intake was 27.9 ± 1.3 μg/kg body weight (BW)/day in the AEGF-C group and 27.4 ± 0.7 μg/kg BW/day in the AEGF-E group. In the PEGF-E group, the EGF intake was 25.0 ± 0.3 μg/kg BW/day for two weeks before ethanol feeding.

### 2.2. Final BW, Relative Liver Weight, and Muscle Weight

The final BW was significantly decreased in the rats in the E, PEGF-E, and AEGF-E groups (Table 2). The liver and relative liver weights increased dramatically in the E group compared with the C group but significantly decreased in the PEGF-E group (Table 2).

### 2.3. Liver Damage

#### 2.3.1. Plasma Aspartate Aminotransferase and Alanine Aminotransferase Activities and Ammonia Concentration

The E group exhibited the highest plasma aspartate aminotransferase (AST) and alanine aminotransferase (ALT) levels, as well as an increase in plasma ammonia concentration (Table 3). Conversely, all EGF intervention groups exhibited decreased plasma AST and ammonia levels compared with the E group (Table 3).

#### 2.3.2. Hepatic TG and TC Levels

As shown in Table 3, the E group represented significantly higher hepatic TG and TC levels when compared to the C group. However, hepatic TG level was significantly decreased in PEGF-C and AEGF-E groups. There was no difference in hepatic TC levels among E, PEGF-C and AEGF-E groups. 

#### 2.3.3. Hepatic Histopathology

**Figure 1:**
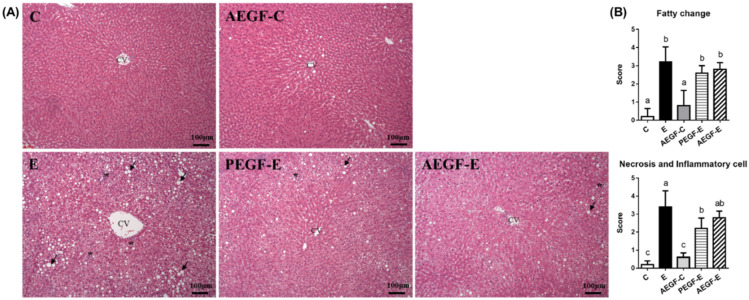
Effect of EGF on liver histopathology in rats fed with ethanol for six weeks (*n* = 6). (**A**) is the histopathological photo and (**B**) is the evaluation score. Values are means ± SDs. Bars with different letters (a, b, c) significantly differ from others, as determined using a one-way analysis of variance with Duncan’s post hoc test (*p* < 0.05). An asterisk (*) indicates the inflammatory cell infiltration site, and an arrow indicates fatty droplets: CV, central vein. The C group was fed a control liquid diet for eight weeks; the E group was fed a control liquid diet for two weeks and then an ethanol-containing diet for six weeks; the AEGF-C group was fed an EGF-containing control liquid diet for eight weeks; the PEGF-E group was fed an EGF-containing control liquid diet for two weeks and then an ethanol-containing liquid diet for six weeks; the AEGF-E group was fed an EGF-containing control liquid diet for two weeks and then an EGF-containing ethanol liquid diet for six weeks.

The representative images of each group are presented in Figure 1A. According to the semiquantitative scoring, the E group had more severe fatty changes, necrosis, and inflammatory cells than the C group. Necrosis and the number of inflammatory cells were significantly decreased in the PEGF-E group compared with the E group. In contrast, the AEFG-E group exhibited a decreasing trend in necrosis and inflammatory cell numbers.

#### 2.3.4. Hepatic Cytokines and TLR4 Signaling Pathway

**Figure 2:**
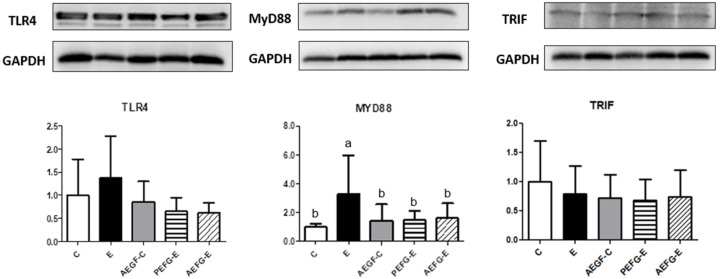
Effect of EGF on TLR4–Myd88/TRIF protein levels in rats fed with ethanol for six weeks. Values are expressed as means ± SDs. Bars with different letters (a, b) significantly differ from others, as determined using a one-way analysis of variance, followed by Duncan’s post hoc test (*p* < 0.05). The groups were the same as those described in Figure 1. TLR4, Toll-like receptor 4; Myd88, myeloid differentiation primary response 88; TRIF, TIR-domain-containing adapter-inducing interferon-β.

The hepatic interleukin (IL)-1β level was significantly increased in the E group than in the C group (Table 4). The PEGF-E and AEGF-E groups exhibited decreased hepatic IL-6 and IL-1β levels compared with the E group (Table 4). The protein level of TLR4 did not differ among the groups (Figure 2). However, the E group exhibited significantly increased hepatic Myd88 protein level but no change in hepatic TIR-domain-containing adapter-inducing interferon-β (TRIF) protein level (Figure 2). The AEGF-C, PEGF-E, and AEGF-E groups revealed a decreased hepatic MyD88 protein level compared to the E group (Figure 2).

### 2.4. Intestinal Injury

#### 2.4.1. Endotoxin and Tight Junctions

**Figure 3:**
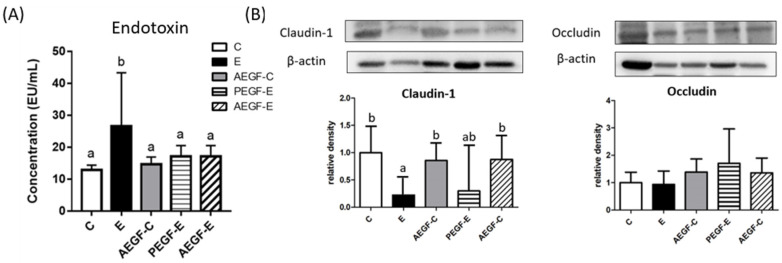
Effect of EGF on endotoxin and tight junction level in rats fed with ethanol for six weeks. (**A**) Endotoxin concentration. (**B**) Protein level. Values are expressed as means ± SDs. Bars with different letters (a, b) significantly differ from others, as determined using a one-way analysis of variance, followed by Duncan’s post hoc test (*p* < 0.05). The groups were the same as those described in Figure 1.

The serum endotoxin level was significantly increased in the E group but significantly decreased in the PEGF-E and AEGF-E groups (Figure 3A). The protein levels of claudin-1 as the intestinal integrity indicators were significantly lower in the E group than in the C group. However, only the AEGF-E group exhibited higher claudin-1 protein levels when compared to the E group (Figure 3B).

#### 2.4.2. Fecal Microbial Diversity and Composition

##### Diversity Indices

**Figure 4:**
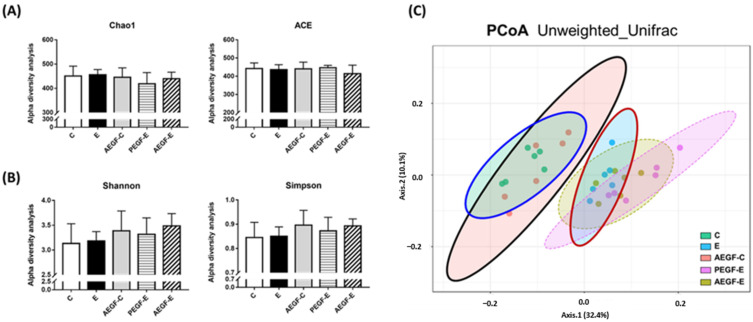
Effect of EGF on the fecal microbiota composition in rats fed with ethanol for six weeks (*n* = 6). (**A**) The richness of fecal microbiota. (**B**) Diversity of fecal microbiota. (**C**) Principal coordinate analysis (PCoA) of fecal microbiota. Values are expressed as means ± SDs and were statistically analyzed through a one-way analysis of variance followed by Duncan’s post hoc test (*p* < 0.05). The areas in (**C**) marked in blue, black, and red represent the composition distribution in the C, AEGF-C, and E groups, respectively. The groups were the same as those described in Figure 1.

The results of the α-diversity indices are presented in Figure 4A,B. The Chao-1 and ACE indices indicate species richness, whereas the Shannon and Simpson indices indicate differences in species. No change in the α-diversity indices was noted among the groups. Figure 4C presents the β-diversity results, which were determined by performing a principal coordinate analysis. The microbiotic compositions could be separated into two groups: control liquid diet (C and AEGF-C) and ethanol-containing liquid diet (E, PEGF-E, and AEGF-E), based on diet. The distribution of the PEGF-E and AEGF-E groups slightly differed from that of the E group.

##### Linear Discriminant Analysis of the Effect Size

From a taxonomic perspective, nine bacterial phyla, 17 classes, 35 orders, 63 families, and 96 genera were identified in this study. We compared the differences in the levels among all groups.

**Figure 5:**
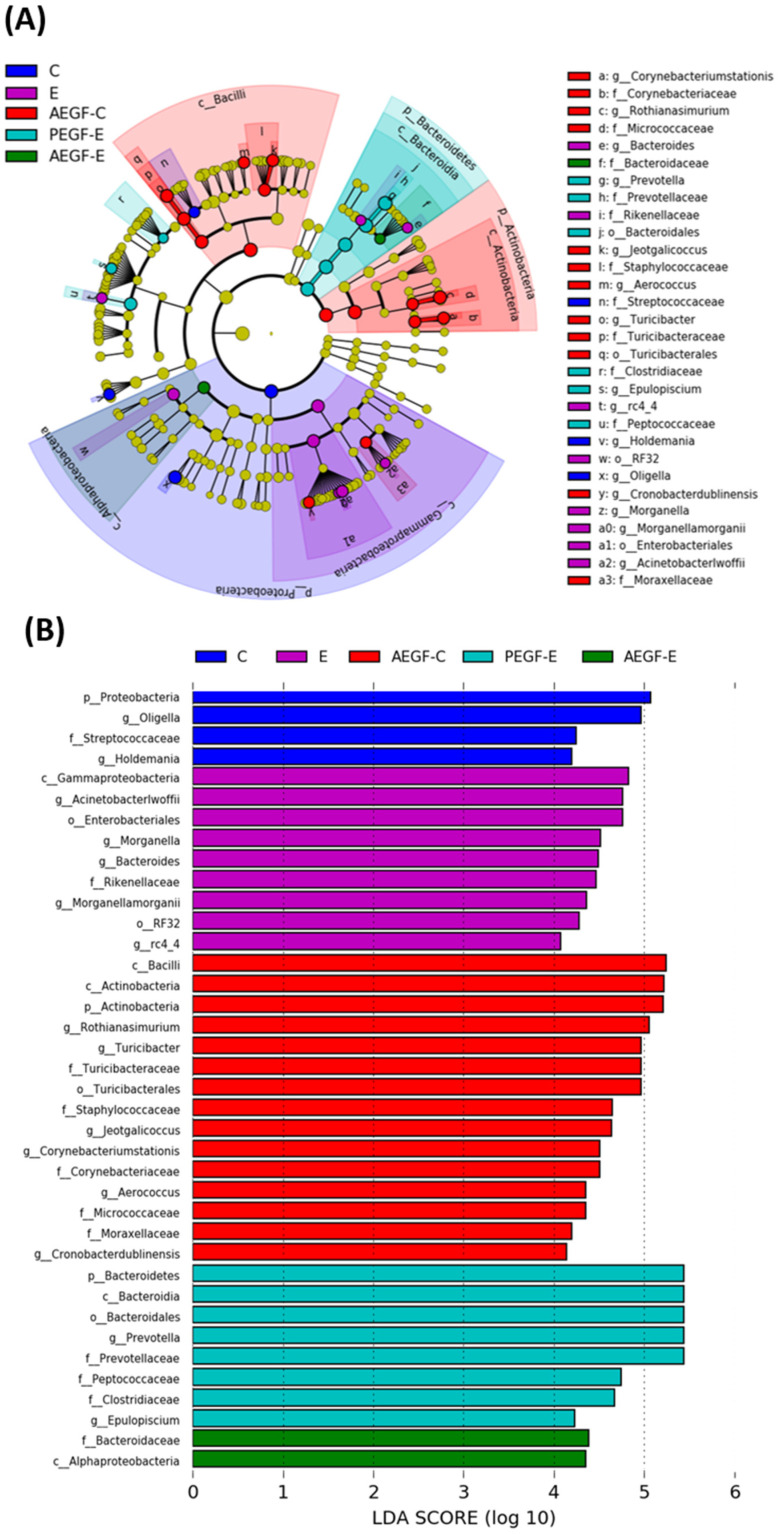
Effect of EGF on fecal microbial taxonomy in rats fed with ethanol for six weeks (*n* = 6). (**A**) Linear discriminant analysis of the effect size (LEfSe). (**B**) Microbiomes in different taxonomical levels above the linear discriminant analysis (LDA) threshold (scores ≥ 4). The groups were the same as those described in Figure 1.

We performed linear discriminant analysis and effect size measurement to analyze the microbial content (Figure 5). Proteobacteria (phylum), Streptococcaceae (family), *Oligella* (genus), and *Holdemania* (genus) were abundant in the C group. Gammaproteobacteria (class), Enterobacteriales (order), RF32 (order), Rikenellamorganii (family), *Actinetobacter iwoffi* (genus), *Morganella* (genus), *Bacteroides* (genus), *Morganella morganii* (genus), and rc4_4 (genus) were abundant in the E group. Actinobacteria (phylum), Bacilli (class), Actinobacteria (type), Turicibacterales (order), Turicbacteraceae (family), Staphylococcaceae (family), Corynebacteriaceae (family), Micrococcaceae (family), Moracellaceae (family), *Rothia nasimurium* (genus), *Turicibacter* (genus), *Jeotgalicoccus* (genus), *Corynebacterium stationis* (genus), *Aerococcus* (genus), and *Cronobacter dublinensis* (genus) were abundant in the AEGF-C group. Bacteroidetes (phylum), Bacteroidia (class), Bacteroidales (order), Prevotellaceae (family), Peptococcaceae (family), Clostridiaceae (family), *Prevotella* (genus), and *Epulopiscium* (genus) were abundant in the PEGF-E group. Alphaproteobacteria (class) and Bacteroidaceae (family) were abundant in the AEGF-E group.

### 2.5. Muscle Loss

#### 2.5.1. Muscle Mass and Grip Strength

The weights of the quadriceps and gastrocnemius are listed in Table 5. No significant differences in the weights of the quadriceps and gastrocnemius were noted among the groups. Grip strength was lower in the E group than in the C group. After EGF supplementation (PEGF-E and AEGF-E groups), the grip strength recovered.

#### 2.5.2. Muscle Histopathology

**Figure 6:**
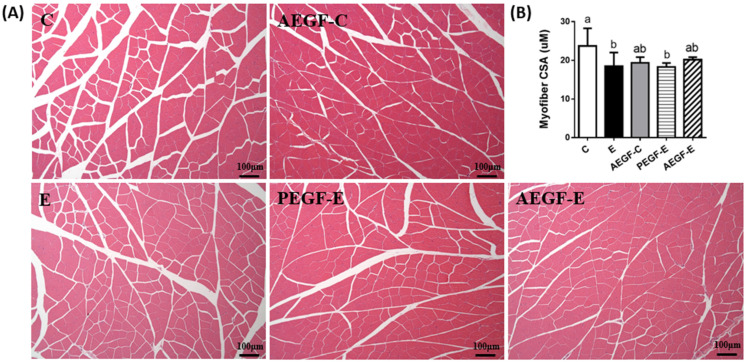
Effect of EGF on muscle histopathology and cross-sectional area (CSA) in rats fed with ethanol for six weeks (*n* = 6). (**A**) Representative hematoxylin and eosin (H&E)-stained histopathological images of the gastrocnemius. (**B**) Quantified analysis of the myofiber CSA. Values are expressed as means ± SDs. Bars with different letters (a, b) significantly differ from others, as determined using a one-way analysis of variance followed by Duncan’s post hoc test (*p* < 0.05). The groups were the same as those described in Figure 1.

The muscle cross-sectional area (CSA) revealed the area of a single myofiber, and quantitative results are shown in Figure 6. The CSA significantly decreased in the E and PEGF-E groups. After eight weeks of EGF supplementation, the CSA also decreased.

#### 2.5.3. Muscular Protein Metabolism

**Figure 7:**
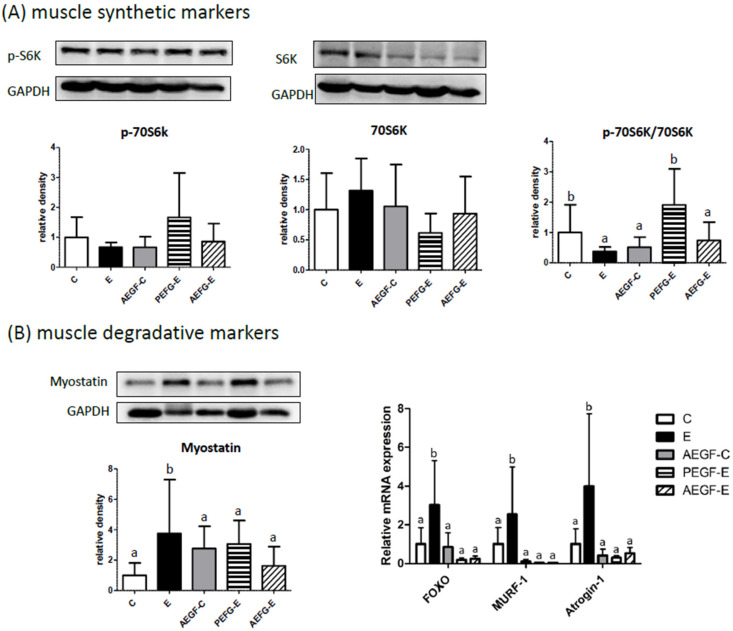
Effects of EGF on muscle synthesis and degradation markers in rats fed with ethanol for six weeks. (**A**) protein levels of muscle synthetic markers. (**B**) protein and mRNA levels of muscle degradative markers. Values are expressed as means ± SDs. Bars with different letters (a, b) significantly differ from others, as determined using a one-way analysis of variance, followed by Duncan’s post hoc test (*p* < 0.05). The groups were the same as those described in Figure 1. 70S6K, ribosomal protein S6 kinase; FOXO, forkhead box transcription factors; MURF-1, myostatin potentiates muscle RING-finger protein-1.

Figure 7A presents the regulators of muscular protein synthesis and degradation. The E group had a lower protein ratio of p70S6K and 70S6K, the protein synthesis factor, than the C group. An increased protein ratio of p70S6K and 70S6K was observed only in the PEGF-E group. However, myostatin protein levels were significantly increased in the E group compared with the C group (Figure 7B). However, compared with the E group, myostatin protein level was significantly inhibited in the AEGF-C, PEGF-E and AEGF-E groups (Figure 7). In addition, the mRNA levels of FOXO, MURF-1 and Atrogin-1 were significantly increased in the E group and decreased in the AEGF-C, PEGF-E and AEGF-E groups (Figure 7).

#### 2.5.4. Amino Acid Profile

The distributions of amino acids in the rats' plasma, liver, and muscles are presented in Supplementary Tables S1–S3. In the plasma, no significant difference in the distributions of amino acids was noted between the C and E groups or between the C and AEGF-C groups (Supplementary Table S1). Compared with the E group, the AEGF-E group had significantly higher valine, isoleucine, total branched-chain amino acids (BCAAs), tyrosine, and phenylalanine levels (Supplementary Table S1). Hepatic lysine was significantly higher in the E group than in the C group (Supplementary Table S2). However, the PEGF-E and AEGF-E groups exhibited significantly lower hepatic lysine levels than the E group (Supplementary Table S2). In the muscle tissues, the leucine, arginine, and histidine levels were significantly increased in the E group compared with the C group. In addition, the PEGF-E group exhibited higher valine, tryptophan, and methionine levels than the E group. In contrast, the AEGF-E group had higher valine, leucine, total BCAA, tryptophan, and methionine levels.

## 3. Discussion

### 3.1. Intakes of Food, Ethanol, and EGF

No differences in food intake levels and total calories were observed among the groups (Table 1). The ethanol intake in the ethanol-fed group was 1.06–1.18 g/100 g BW/day. This converted to the human equivalent is a daily alcohol intake of approximately 98.3–103.2 g. This is considered heavy alcohol use, as defined by the National Institute on Alcohol Abuse and Alcoholism (NIAAA) [21]. The EGF intake was similar between the AEGF-C and AEGF-E groups. In addition, before ethanol feeding, the EGF intake in the PEGF-E group was approximately 25 μg/kg BW/day.

### 3.2. BW and Relative Liver Weight

Similar to the findings of our previous studies, the final BW was significantly decreased in the rats fed an ethanol-containing liquid diet, which was reflected in the food efficiency (Table 2) [20,22]. Ethanol provides calories without macronutrients and micronutrients and interferes with the gastrointestinal system, causing malabsorption and dysbiosis [23]. Lipid droplet accumulation causes hepatocyte swelling during the early stages of ethanol-induced pathogenesis [2]. This might explain the significant increase in liver weight in the ethanol-fed groups (Table 2).

### 3.3. Liver Damage and EGF Supplementation

The plasma AST, ALT activities and ammonia levels were significantly increased after 6-week ethanol administration without EGF supplementation (C vs. E group, Table 3). In addition, the higher hepatic TG and TC levels and H&E staining scores indicated that the hepatic tissue exhibited evident fatty changes and inflammatory cell accumulation in the E group, which is in accordance with the activation of Myd88 protein and the elevation of the higher hepatic IL-1b level (Figure 1 and Figure 2, Table 3 and Table 4). Alcohol and its metabolite acetaldehyde impede hepatic ureagenesis and cause hyperammonemia [24]. Moreover, reactive oxygen species and oxidative stress are elevated when proinflammatory cytokines are overexpressed [25]. The present study’s findings suggest that ethanol-induced liver damage was successfully established in the E group, as reported in our previous studies [20,26].

A higher serum endotoxin level was observed in the E group (Figure 3A). Endotoxins translocated from the leaky gut by the interruption of alcohol and acetaldehyde activated the TLR4 pathway, which further activated downstream and eventually recruited cytokines [27,28]. Although the TLR4 protein level did not change in this study, the hepatic Myd88 protein level significantly increased (Figure 2). TLRs play crucial roles in the innate immune system in that they recognize pathogen-associated molecular patterns derived from various microbes, such as LPSs [29]. After TLR engagement, Myd88 and TRIF proteins are activated simultaneously, resulting in varying degrees of inflammatory responses. Myd88 protein induces the production of cytokines with strong inflammatory responses, and TRIF protein promotes the generation of interferons with weak inflammatory responses [30]. Therefore, long-term alcohol intake might induce strong inflammatory responses via the Myd88 route and thereby cause alcoholic hepatitis.

When EGF was provided to rats before they were fed an ethanol-containing liquid diet, their hepatic inflammation and IL-1b levels significantly decreased; however, no hepatic fatty changes were observed (Figure 1B, Table 4, E vs. PEGF-E group). On the other hand, a significantly lower hepatic TG level was found in rats supplemented with EGF during an ethanol-containing liquid diet (Table 3, E vs. AEGF-E group). Furthermore, the plasma AST activity, ammonia, and serum endotoxin levels significantly decreased in both the PEGF-E and AEGF-E groups (Table 3, Figure 3A). In addition, the higher MyD88 protein level was significantly suppressed in the EGF supplementation groups (Figure 2, E vs. PEGF-E; E vs. AEGF-E group). Therefore, it was assumed that EGF supplementation reduced ethanol-induced liver inflammation by inhibiting the translocation of LPSs from the gut. Based on this phenomenon, we investigated intestinal damage related to intestinal tight junction proteins and microbiota composition.

### 3.4. Intestinal Damage and EGF

The rats fed an ethanol-containing liquid diet for six weeks (E group) exhibited significantly lower claudin-1 and occludin protein levels, which might represent damaged tight junctions (Figure 3B). This result is consistent with the higher serum endotoxin level observed in the E group (Figure 3A). In the intestinal mucosa, epithelial tight junctions provide a diffusion barrier that prevents toxic agents, allergens, and pathogens from reaching tissues and systemic circulation [31,32]. Alcohol treatment reduced occludin and claudin-1 mRNA expression [33,34]. Additionally, long-term alcohol consumption altered the microbiotic composition of the intestine and disrupted the immune system through ethanol and acetaldehyde production [5]. Thus, long-term alcohol consumption impairs intestinal tight junction proteins, allowing endotoxins to enter the bloodstream. This study demonstrated that EGF supplementation inhibited the reduction of claudin-1 protein level and the elevation of the serum endotoxin level induced by chronic ethanol consumption (Figure 3B). EGF regulates cell growth, survival, migration, apoptosis, proliferation, and differentiation [13]. In addition to enhancing cellular proliferation and differentiation, EGF functions as a gastrointestinal tract mucosal protective factor and is associated with intestinal maturation and maintenance of epithelial cell homeostasis in the small intestine [35]. A previous study indicated EGF treatment reduced morbidity in septic mice by maintaining the homeostasis of intestinal apoptosis and proliferation and restoring gut integrity [36]. Therefore, EGF supplementation protects the intestinal mucosa against the damage caused by long-term ethanol consumption.

In the present study, no differences were noted in a-diversity among the groups (Figure 4). This result is consistent with those of our previous studies [22,26,37]. However, compared with other studies, the ratio of fecal firmicutes to bacteroidetes and a-diversity in the ethanol-fed animals of the present study was not concordant because of differences in the administration routes, feeding durations, and animal species [38,39,40].

In the E group, more gram-negative and pathogenic bacteria were found in the fecal microbiota (Figure 5). Gammaproteobacteria were determined to be abundant in children with NAFLD and to interfere with short-chain fatty acid (SCFA) production [41]. In addition, increased Enterobacteriales were proposed to be a characteristic of a leaky intestine [42]. RF32 was positively associated with bowel inflammation [43] and raised in mice with ethanol poisoning [44]. Clostridiaceae, an SCFA bacterial producer, decreased in viral chronic liver diseases [45]. Prevotellaceae and Prevotella were positively associated with alcohol intake in patients with cirrhosis [46] and were translocated from the intestinal mucosal layer to the liver [40].

Furthermore, *A. lwoffi* is a cause of nosocomial infections, potentially resulting in gastroenteritis. It was found in patients with irritable bowel syndrome, small intestine bacterial overgrowth, and liver abscess [47,48,49]. The present study indicated that rats fed an ethanol-containing diet for a long period had higher levels of proinflammatory bacteria in the gut that produced endotoxins.

A meta-analysis of nine studies reported that the Bacteroidia class was decreased in rodents with high-fat-diet-induced obesity. In the present study, this class was relatively abundant in the PEGF-E group (Figure 5) [50]. Additionally, a study in 2018 reported that the genus belonging to the Bacteroidaceae family was decreased in rats continually fed with ethanol [51]. Bermingham et al. indicated that Clostridiaceae is the central node in the relationship among microbiota, macronutrient composition and digestibility, and fecal health score and weight [52]. In contrast to increasing diversity, EGF supplementation can be considered to have changed the microbiota composition in the rats fed an ethanol-containing liquid diet.

### 3.5. Muscular Protein Metabolism and EGF Supplementation

In the present study, the ratio of p-70S6K/70S6K as the muscular protein synthesis factor was significantly decreased in the E group (C vs. E group, Figure 7A). Moreover, as the muscular protein degradation factors, the protein level of myostatin and mRNA levels of FOXO, MURF, and atrogin-1 were significantly elevated in the E group (C vs. E group, Figure 7B). Myoblast fusion is required for muscle growth and regeneration and is inhibited by myostatin [53,54]. A clinical trial revealed that heavy alcohol drinkers had a higher risk of sarcopenia [55]. Similarly, alcohol-fed rats have been reported to undergo a loss of muscle mass and to exhibit increased muscular autophagy and myostatin protein levels [56,57].

Furthermore, higher MURF-1 and antrogin-1 have been observed in alcohol-fed animals [56,58]. The present results are in accordance with previous studies indicating that long-term ethanol intake induced myostatin protein levels. In addition, high plasma endotoxin and ammonia levels have been reported to reduce the Akt and mTOR protein levels and increase myostatin and MURF-1 levels, which may contribute to the increased degradation of muscle proteins [59,60,61]. In the present study, the rats fed with ethanol had higher levels of serum endotoxin (intestinal damage) and plasma ammonia (liver damage), possibly related to muscle loss due to long-term ethanol consumption. Furthermore, grip strength and myofiber CSA significantly decreased in the E group (C vs. E group, Figure 6), indicating the rats fed with ethanol had weakened muscles.

EGF supplementation significantly inhibited the elevation of muscular myostatin protein levels in the rats fed with ethanol (E vs. PEGF-E and AEGF-E groups, Figure 7B). As the downstream factors of muscular degradation, EGF supplementation also significantly decreased the mRNA levels of FOXO, MURF-1, and atrogin-1 (E vs. PEGF-E and AEGF-E groups, Figure 7B). EGF supplementation might positively affect maintaining muscle mass, which was also associated with lower serum endotoxin and plasma ammonia levels in the rats fed with ethanol and EGF. EGF, involved in satellite cell proliferation, is crucial for muscle mass maintenance [62,63]. After EGF binds to its receptor (EGFR), it controls cell proliferation and differentiation through the phosphoinositide-dependent kinase-1/Akt/mTOR/p70S6K pathway to conduct protein synthesis [64]. Vuorela et al. reported that the serum EGF level was diminished by alcohol abuse in pregnant women [65]. However, in the present study, other biomarkers of muscle loss, such as muscle CSA and grip strength, did not significantly differ among the E, PEGF-E, and AEGF-E groups (Table 5). Future studies should include a longer experimental period.

### 3.6. Amino Acid Composition

We analyzed the amino acid profiles in the plasma, liver, and muscles (Supplementary Tables S1–S3), which contribute to the kinetic balance of body amino acids. A greater shift in amino acids was observed in the plasma and muscle than in the liver. Several factors can affect plasma amino acid composition, including long-term alcohol consumption, dietary protein deficiencies, and liver disease severity [66]. Plasma BCAA levels may be abnormal in liver cirrhosis and result in muscle protein degradation [62,67]. Long-term alcohol consumption in baboons produced higher plasma BCAA levels due to impaired metabolism of BCAAs in muscles [66]. In the present study, EGF supplementation might have promoted the dynamic effects of BCAAs on muscle synthesis, although no effects of long-term alcohol consumption on BCAA levels were observed.

A higher hepatic lysine level was found in the E group (C vs. E group, Supplementary Table S2). However, the hepatic lysine level was significantly decreased in the PEGF-E and AEGF-E groups (E vs. PEGF-E and AEGF-E group, Supplementary Table S2). Shepard and Tuma reported that long-term ethanol consumption induced hepatic lysine hyperacetylation, which was related to several metabolic factors, including p53, sterol response element binding protein-1c, peroxisome proliferator-activated receptor g coactivator a, acetyl CoA synthetase 2, and tubulin [68]. Future studies should determine whether lysine hyperacetylation can explain the higher hepatic lysine level in the rats fed with ethanol in the present study.

### 3.7. Study Limitation

This study has several limitations that should be considered. To clarify the relationship between muscle strength and muscle loss, the muscle weight of the forelimbs should also be measured in the experiment. Moreover, the alteration of muscle metabolism and the signaling pathway under long-term ethanol intake was incompletely illustrated. The muscular protein synthesis factors should be confirmed, such as 4EBP1. The muscular TLR pathway, autophagy pathway or anti-inflammatory markers responding to decreased endotoxin levels should be investigated in future studies. In addition, other factors exerting diverse effects on pre-administration (PEGF-E group) and administration along with ethanol induction (AEGF-E group) should be determined. EGF supplementation for progressive ALD and other optional beneficial dosages should be discussed in further investigations.

## 4. Materials and Methods

### 4.1. Animals and Study Protocol

**Figure 8:**
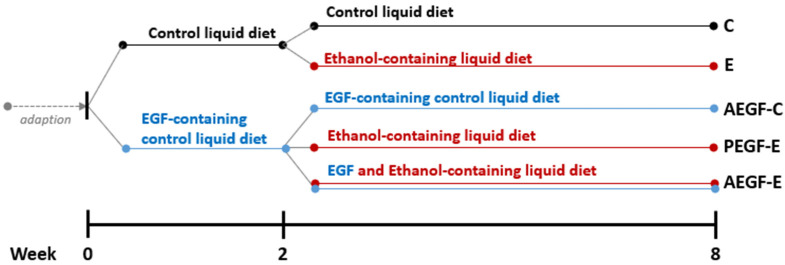
The animals, diets, and groups. The C group was fed a control liquid diet for eight weeks; the E group was fed a control liquid diet for two weeks and then an ethanol-containing diet for six weeks; the AEGF-C group was fed an EGF-containing control liquid diet for eight weeks; the PEGF-E group was fed an EGF-containing control liquid diet for two weeks and then an ethanol-containing liquid diet for six weeks; the AEGF-E group was fed an EGF-containing control liquid diet for two weeks and then an EGF-containing ethanol liquid diet for six weeks.

All study procedures were approved by the Institutional Animal Care and Use Committee of Taipei Medical University (LAC-2017-0384). Fifty 6-week-old Wistar rats (BioLasco Taiwan, Ilan, Taiwan) were individually housed in a room with 50–70% humidity and a 12-h light–dark cycle. All the rats were fed a control liquid diet for one week during the acclimation period. Blood samples were drawn from the tail vein, and AST and ALT activities were measured. The rats were divided into five groups based on their AST and ALT activities. Based on our previous study, the small-intestine mucosal EGF content in the E group was significantly higher than indicated damage of small-intestine mucosa [20]. Therefore, based on the viewpoint of prevention, we decided to pre-treat EGF to investigate the prevention effects of EGF on chronic alcoholic damage. As shown in Figure 8, two groups were fed a control liquid diet, and three groups were fed an EGF-containing liquid diet for two weeks. Subsequently, the control diet groups were fed a control liquid diet (C) or ethanol-containing liquid diet (E) for six weeks. Similarly, among the three EGF-containing liquid diet groups, one was continually fed the same diet (AEGF-C); the diets of the other two groups were changed to an ethanol-containing liquid diet without EGF (PEGF-E) and an ethanol-containing liquid diet with EGF (AEGF-E). The composition of the liquid diet is described in Supplementary Table S4 and was determined based on a previous study [20]. Daily diet consumption and BW were measured routinely, and the dosage of EGF was 30 mg/kg BW/day in accordance with the weekly BW measurement [20]. After eight weeks, all rats were anesthetized and sacrificed. Blood samples were drawn through the ventral aorta, and the organs were collected for subsequent experiments. 

### 4.2. Assessment of Liver Damage

#### 4.2.1. Liver Function

Blood samples were collected in a sodium-heparin-coated vacutainer (Becton, Dickinson and Company, Franklin Lakes, NJ, USA) and centrifuged at 1200× *g* at 4 °C for 15 min. Liver function markers, AST, and ALT, were measured using the ADVIA Chemistry XPT system (Siemens Healthineers, Eschborn, Germany).

#### 4.2.2. Liver Histological Assessment

The caudate lobe of the liver was fixed in 10% formaldehyde solution immediately after sacrifice, and histological slices were stained with hematoxylin and eosin (H&E). Semiquantitative histological scoring was conducted by a veterinarian unaware of the study design and assigned a grade of 0 to 4 (0 = absent, 1 = trace, 2 = mild, 3 = moderate, and 4 = severe).

#### 4.2.3. Hepatic Triglyceride (TG) and Total Cholesterol (TC)

350–400 mg of the liver was homogenized with 1.5 mL nonyl phenoxypolyethoxylethanol (NP-40) containing protease inhibitor. The homogenate was centrifuged at 10,000× *g* for 10 min at 4 °C. All supernatants, including the lipid layer, were collected, and future procedures were performed according to the manufacturer’s instructions. First, Triglyceride colorimetric assay kit (10010303, Cayman Chemical, Ann Arbor, MI, USA) was used to measure the concentration of triglyceride in the liver. The sample preparation of total cholesterol determination was based on the protocol provided by the manufacturer. Next, 50 mg of liver tissue was homogenized by 1 mL solvent (chloroform:isopropanol:NP-40 of 7:11:0.1) and centrifuged at 15,000× *g* for 10 min at 4 °C. 200 μL of supernatant was collected in a new tube. Air dry at 50 °C to remove the chloroform, then put samples under a vacuum to remove the trace amounts of organic solvent. Dried lipids were dissolved in 200 μL of kit provided assay diluent. The extract was further analyzed with a cholesterol colorimetric assay kit (Cell Biolabs, San Diego, CA, USA).

#### 4.2.4. Hepatic Cytokines

Hepatic tissues were homogenized with three volumes of buffer containing 150 mM NaCl, 50 mM Tris-HCl, 0.1% sodium dodecylsulfate (SDS), and 1% Triton X-100. The homogenates were centrifuged at 3000× *g* and 4 °C for 15 min. Then, the supernatants were collected for the subsequent analysis. Inflammatory cytokines, including IL-1β, IL-6, IL-10, and tumor necrosis factor-α, were detected using commercial enzyme-linked immunosorbent assay kits (dy501, dy506, dy522, and dy510; R&D Systems, Minneapolis, MN, USA).

#### 4.2.5. Hepatic Inflammatory Protein Level

We homogenized 0.1 g of the hepatic tissue with four volumes of RIPA buffer containing 1% PI and then conducted centrifugation at 3000× *g* and 4 °C for 15 min. The supernatants were collected. The TLR4–MyD88 proinflammatory pathway and TRIF levels were detected through western blotting. The antibodies that were used are listed in Supplementary Table S5.

### 4.3. Intestinal Health Status Examination

#### 4.3.1. Tight Junction Protein Level

We homogenized 50 mg of the ileal tissue with RIPA buffer containing 1% PI and then conducted centrifugation at 3000× *g* and 4 °C; the supernatants were collected. Protein separation was conducted through sodium dodecylsulfate polyacrylamide gel electrophoresis (SDS-PAGE). Claudin-1 and occludin were detected using the antibodies listed in Supplementary Table S5. Proteins were electroblotted onto a polyvinylidene difluoride (PVDF) transfer membrane and then incubated with antibodies, and β-actin was used as an internal control. Finally, the blot was treated with anti-rabbit IgG. Bands were quantified using Image-Pro Plus 4.5 software.

#### 4.3.2. Microbiota Analysis

Metagenomic studies are commonly performed by analyzing the prokaryotic 16S ribosomal RNA gene (16S rRNA). Variable 16S rRNA regions are frequently used in phylogenetic classifications, such as genus or species classifications, in diverse microbial populations. We used 2.5 μL of DNA to set up the first polymerase chain reaction (PCR) with 0.2 μM V3+V4 forward and reverse primers (Forward: TCGTCGGCAGCGTCAGATGTGTATAAGAGACAGCCTACGGNGGCWGCAG, Reverse: GTCTCGTGGGCTCGGAGATGTGTATAAGAGACAGGATACHVGGGTATCTAATCC) and 12.5 μL of 2× Kapa HiFi HotStart ReadyMix (KapaBiosystems, Wilmington, MA, USA) in 25-μL reactions. The PCR cycling conditions were 3 min at 95 °C and 25 cycles of 30 s at 95 °C, 30 s at 55 °C, and 30 s at 72 °C followed by 5 min at 72 °C. The amplified DNA was purified using Agencourt AMPure XP Reagent beads (Beckman Coulter, Brea, CA, USA). The second PCR was set up to add indexes to the amplified DNA by adding 5 μL of purified DNA to 25 μL 2× Kapa HiFi HotStart ReadyMix (KapaBiosystems) and 5 μL of Nextera XT Index 1 and 2 primers (Illumina, San Diego, CA, USA) in 50-μL reactions. The second PCR reaction was set at 3 min at 95 °C and 8 cycles of 30 s at 95 °C, 30 s at 55 °C, and 30 s at 72 °C followed by 5 min at 72 °C. The amplified DNA was purified using Agencourt AMPure XP Reagent beads purification (Beckman). We used qPCR (KAPA SYBR FAST qPCR Master Mix) to quantify each library using Roche LightCycler 480 system. We pooled them to obtain 4 nM for the Illumina MiSeq NGS system (Illumina, San Diego, CA, USA). More than 100,000 reads with paired-end sequencing (2 × 300 bp) were generated.

### 4.4. Muscle Mass and Related Protein Level

#### 4.4.1. Grip Strength

At baseline and the eighth week, the forelimb grip strength was measured by an animal forelimb grip strength measuring device (Model-RX-5, Aikoh Engineering, Nagoya, Japan).

#### 4.4.2. Histological Examination and Muscle CSA

The right side of the gastrocnemius, which was cut from the middle, was fixed in a 10% formaldehyde solution immediately after removal from the rats. With H&E staining, the CSA was quantized using Image-Pro Plus Software 4.5 (Media Cybernetics, Rockville, MD, USA).The CSA (mm) was calculated using the following formula: area (mm)/the amount of myofiber (under 100× field of view). 

#### 4.4.3. Protein Level of Muscular Protein Synthesis and Degradation Factors

Right gastrocnemius muscle tissue (0.1 g) was homogenized in 0.4 mL RIPA buffer (50 mM Tris-HCl, 150 mM NaCl, 0.1% SDS, and 1% NP-40 at pH 7.5) containing 1% of a PI (HYK0010, MedChemExpress, Monmouth Junction, NJ, USA) and phosphatase inhibitor (PPI) (HYK0022, MedChemExpress, Monmouth Junction, NJ, USA). After an ice bath for 30 min, the homogenate was centrifuged at 10,000× *g* for 10 min at 4 °C. The target proteins were analyzed by western blotting, described as the tight junction protein level measurement. The antibodies are listed in Supplementary Table S5.

#### 4.4.4. mRNA Expression of Muscular Protein Synthesis and Degradation Factors

TRI Reagent (Sigma-Aldrich, St. Louis, MO, USA) was used to isolate and extract the RNA of the left gastrocnemius muscle in accordance with the manufacturer’s instructions. Total RNA was reverse transcribed using the RevertAid First Strand cDNA Synthesis kit (#K1621, Thermo Fisher Scientific, Waltham, MA, USA). The primer sequences for analyzing mRNA were listed in Supplementary Table S6. The resulting cDNA was amplified in a 96-well PCR plate with SYBR Green/ROX qPCR Master Mix (2×, Thermo Fisher Scientific) on a QuantStudio 1 Real-Time PCR System (Thermo Fisher Scientific). Gene levels were normalized to the GAPDH level, and all groups were compared with the C group by setting the C group to 1 [22,69]. 

### 4.5. Plasma, Hepatic, and Muscular Amino Acids Profiles

Using the protocol described by Yuan et al. [70], amino acids were extracted from the mice's plasma, liver, and muscle tissues. Amino acid analysis was performed through ultraperformance liquid chromatography (UPLC) (Acquity UPLC System, Waters, Milford, MA, USA) coupled with an Xevo TQ MS column (Waters, Milford, MA, USA). For the UPLC, a 1.7-mm (2.13100 mm) C18 column (Acquity UPLC System; Waters) was used. Liquid chromatography separation was performed at 40 °C at a flow rate of 0.3 mL/min by using the gradient for the analysis as follows: 0 to 0.5 min 1% B, 0.5 to 2.5 min from 1% B to 10% B, 2 to 3.5 min from 10% to 35% B, 3.5 to 6 min from 35% to 99% B, and 6 to 9 min 1% B [solvent system A: water/formic acid (100:0.1, *v*/*v*); B: acetonitrile/formic acid (100:0.1, *v*/*v*)]. TargetLynx Software 4.1(Waters, Milford, MA, USA) were used to acquire data.

### 4.6. Chemiclas and Kits 

All chemicals and kits used in this study were listed in Supplementary Table S7.

### 4.7. Statistical Analysis

Values are presented as means ± SDs. Statistical differences among the groups were determined using the *t* test or one-way analysis of variance and Duncan’s multiple-range test. All statistical analyses were performed using SAS software 9.4 (SAS Institute, Cary, NC, USA). A *p* value of <0.05 indicated a significant difference.

## 5. Conclusions

Despite no apparent improvement in muscle loss, EGF supplementation significantly increased grip strength. It inhibited the elevated muscular protein level of myostatin and mRNA level of FOXO, MUFR-1 and atrogin-1, which played a crucial role in protein degradation in the muscles of rats fed an ethanol-containing liquid diet. Two significant pathways might be responsible for reducing muscular myostatin caused by EGF supplementation. First, intestinal tight junctions might be strengthened, and a healthy microbiota composition may be maintained by EGF, which inhibits endotoxins from entering the bloodstream. Second, EGF supplementation might improve the hepatic metabolic ability, thus reducing plasma ammonia levels and increasing BACC levels in muscle tissues. However, it is necessary to confirm the reproducibility of the results in future studies.

## Figures and Tables

**Table 1:** Effect of EGF on the intake of food, ethanol, and EGF in rats fed with ethanol for six weeks ^1,2,3^.

Groups	Food Intake (g/100 g BW/Day)	Ethanol Intake (g/100 g BW/Day)	Food Efficiency (%)
C	23.7 ± 0.7	-	4.4 ± 0.4 ^b^
E	24.7 ± 0.7	1.14 ± 0.04	3.2 ± 0.6 ^a^
AEGF-C	23.4 ± 1.1	-	4.9 ± 0.6 ^b^
PEGF-E	24.1 ± 0.5	1.10 ± 0.04	3.1 ± 0.4 ^a^
AEGF-E	23.9 ± 0.6	1.09 ± 0.03	3.5 ± 0.5 ^a^

^1^ All values are presented as means ± SDs. ^2^ Values with the same letter in a column are not significantly different, as determined using a one-way analysis of variance followed by Duncan’s multiple range test, *p* < 0.05. ^3^ The C group was fed a control liquid diet for eight weeks; the E group was fed a control liquid diet for two weeks and then an ethanol-containing diet for six weeks; the AEGF-C group was fed an EGF-containing control liquid diet for eight weeks; the PEGF-E group was fed an EGF-containing control liquid diet for two weeks and then an ethanol-containing liquid diet for six weeks; the AEGF-E group was fed an EGF-containing control liquid diet for two weeks and then an EGF-containing ethanol liquid diet for six weeks.

**Table 2:** Effect of EGF on the final body weight, liver and relative liver weight, quadriceps, and gastrocnemius in rats fed with ethanol for six weeks ^1,2,3^.

Groups	Final Body Weight (g)	Liver Weight (g)	Relative Liver Weight (%)
C	422.0 ± 28.4 ^b^	10.4 ± 0.8 ^a^	2.5 ± 0.0 ^a^
E	375.8 ± 22.1 ^a^	13.2 ± 1.5 ^c^	3.5 ± 0.5 ^c^
AEGF-C	434.5 ± 25.1 ^b^	10.8 ± 0.9 ^a^	2.5 ± 0.1 ^a^
PEGF-E	364.3 ± 17.1 ^a^	11.3 ± 0.9 ^ab^	3.1 ± 0.2 ^b^
AEGF-E	367.8 ± 32.1 ^a^	12.7 ± 1.6 ^bc^	3.5 ± 0.4 ^c^

^1^ All values are presented as means ± SDs. ^2^ Values with the same letter in a column are not significantly different, as determined using a one-way analysis of variance followed by Duncan’s multiple range test, *p* < 0.05. ^3^ The groups were the same as those described in Table 1.

**Table 3:** Plasma liver function indicators, ammonia, hepatic triglyceride (TG) and total cholesterol (TC) levels in rats fed with ethanol for six weeks ^1,2,3^.

Groups	AST (U/L)	ALT (U/L)	Ammonia (μg/dL)	TG mg/g Liver	TC mg/g Liver
C	78 ± 2 ^a^	38 ± 1 ^a^	104 ± 10 ^b^	49.2 ± 6.5 ^c^	3.01 ± 0.19 ^b^
E	298 ± 93 ^b^	180 ± 83 ^b^	175 ± 40 ^c^	99.9 ± 8.2 ^a^	4.31 ± 0.30 ^a^
AEGF-C	75 ± 2 ^a^	38 ± 2 ^a^	63 ± 7 ^ab^	65.2 ± 7.6 ^bc^	3.49 ± 0.33 ^ab^
PEGF-E	141 ± 11 ^a^	97 ± 7 ^ab^	24 ± 8 ^a^	77.4 ± 10.3 ^ab^	3.47 ± 0.20 ^ab^
AEGF-E	164 ± 20 ^a^	108 ± 10 ^ab^	34 ± 10 ^a^	68.2 ± 8.2 ^bc^	3.66 ± 0.31 ^ab^

^1^ All values are presented as means ± SEMs. ^2^ Values with the same letter in a column are not significantly different, as determined using a one-way analysis of variance followed by Duncan’s multiple range test, *p* < 0.05. ^3^ The groups were the same as those described in Table 1.

**Table 4:** Effects of EGF on hepatic cytokine levels in rats fed with ethanol for six weeks ^1,2,3^.

Groups	TNF-α (pg/mg Protein)	IL-1β (pg/mg Protein)	IL-6 (pg/mg Protein)	IL-10 (pg/mg Protein)
C	16.39 ± 2.86	70.40 ± 13.36 ^ab^	175.29 ± 35.56 ^b^	85.86 ± 21.64 ^b^
E	18.89 ± 2.64	101.21 ± 20.89 ^c^	186.39 ± 35.43 ^b^	110.16 ± 29.05 ^ab^
AEGF-C	19.25 ± 4.40	54.51 ± 15.82 ^a^	114.18 ± 27.65 ^a^	69.28 ± 20.11 ^a^
PEGF-E	18.06 ± 4.50	58.07 ± 15.60 ^a^	87.17 ± 32.12 ^a^	77.69 ± 21.41 ^a^
AEGF-E	16.57 ± 4.02	80.54 ± 15.03 ^b^	76.84 ± 17.57 ^a^	86.80 ± 12.05 ^ab^

^1^ All values are means ± SDs. ^2^ Values with the same letter in a column are not significantly different, as determined using one-way analysis of variance, followed by Duncan’s multiple range test, *p* < 0.05. ^3^ The groups were the same as those described in Table 1.

**Table 5:** Plasma liver function indicators and ammonia levels in rats fed with ethanol for six weeks ^1,2,3^.

Groups	Quadriceps Weight (g)	Gastrocnemius (g)	Grip Strength (g)
C	4.4 ± 1.6	6.9 ± 2.1	1673.31 ± 75.54 ^c^
E	5.9 ± 1.5	5.1 ± 1.2	1350.37 ± 150.88 ^a^
AEGF-C	6.8 ± 1.6	5.6 ± 0.6	1562.29 ± 69.73 ^bc^
PEGF-E	5.4 ± 1.1	4.9 ± 0.4	1572.16 ± 109.75 ^bc^
AEGF-E	5.7 ± 1.5	5.2 ± 1.1	1484.23 ± 105.29 ^b^

^1^ All values are presented as means ± SDs. ^2^ Values with the same letter in a column are not significantly different, as determined using a one-way analysis of variance followed by Duncan’s multiple range test, *p* < 0.05. ^3^ The groups were the same as those described in Table 1.

## Data Availability

The data presented in this study are contained within the article and supplementary materials.

## Supplementary Materials

The supporting information can be downloaded at: https://www.mdpi.com/article/10.3390/ijms24108845/s1.
