# Supplementary material for: Potential Benefits of Epidermal Growth Factor for Inhibiting Muscle Degrative Markers in Rats with Alcoholic Liver Damage
Source: Int J Mol Sci. 2023 May 16;24(10):8845. doi: 10.3390/ijms24108845 (PMC10219006; doi:10.3390/ijms24108845)
Supplement: Supplementary file 1 [file ijms-24-08845-s001.zip › ijms-2339942-supplementary.pdf]

Supplementary Table S1. Effects of EGF on plasma amino acid composition in rats fed with ethanol for 6 weeks[1,2].

Groups[3]	C	E	AEGF-C	PEGF-E	AEGF-E
Valine (μM)	183.35 ± 14.67 ^{b}	187.35 ± 13.16 ^{b}	209.49 ± 42.07 ^{ab}	218.64 ± 27.95 ^{ab}	289.39 ± 100.42 ^{a}
Leucine (μM)	224.82 ± 90.45 ^{b}	288.38 ± 33.13 ^{ab}	404.42 ± 122.26 ^{ab}	295.91 ± 14.84 ^{ab}	528.42 ± 283.28 ^{a}
Isoleucine (μM)	270.09 ± 46.63 ^{b}	297.87 ± 16.06 ^{b}	336.90 ± 86.47 ^{ab}	319.95 ± 6.76 ^{ab}	425.19 ± 90.49 ^{a}
BCAA (μM)	678.25 ± 144.45 ^{b}	773.61 ± 58.00 ^{b}	950.81 ± 187.97 ^{ab}	834.50 ± 25.88 ^{b}	1243.01 ± 395.92 ^{a}
Tyrosine (μM)	164.89 ± 10.54 ^{ab}	146.51 ± 3.90 ^{b}	219.08 ± 119.07 ^{ab}	152.80 ± 12.42 ^{ab}	308.06 ± 136.91 ^{a}
Tryptophan (μM)	24.58 ± 2.35	21.91 ± 1.79	28.41 ± 5.36	22.53 ± 0.99	24.15 ± 12.08
Phenylalanine (μM)	183.34 ± 33.71 ^{b}	166.91 ± 17.33 ^{b}	161.05 ± 10.13 ^{b}	208.66 ± 51.00 ^{ab}	365.14 ± 191.85 ^{a}
AAA (μM)	372.80 ± 34.40	335.33 ± 19.60	408.55 ± 118.88	383.99 ± 40.09	397.35 ± 301.45
Threonine (μM)	0.00 ± 0.00	0.05 ± 0.09	0.07 ± 0.13	0.11 ± 0.14	0.00 ± 0.00
Serine (nM)	0.00 ± 0.00	0.00 ± 0.00	0.00 ± 0.00	0.00 ± 0.00	0.00 ± 0.00
Proline (μM)	26.24 ± 2.48 ^{c}	30.95 ± 2.07 ^{bc}	33.52 ± 0.54 ^{abc}	37.77 ± 0.89 ^{ab}	41.92 ± 9.98 ^{ab}
Lysine (μM)	3.31 ± 2.20	3.01 ± 2.11	4.11 ± 1.86	0.51 ± 1.16	4.86 ± 2.96
Glycine (μM)	0.00 ± 0.00	0.00 ± 0.00	0.00 ± 0.00	0.00 ± 0.00	0.00 ± 0.00
Glutamine (μM)	7.58 ± 0.33 ^{a}	6.47 ± 0.33 ^{ab}	7.03 ± 1.06 ^{a}	3.21 ± 2.27 ^{b}	6.17 ± 3.52 ^{ab}
Glutamic acid (μM)	2.39 ± 0.63	1.80 ± 0.34	2.07 ± 0.28	2.43 ± 0.92	2.67 ± 0.51
Aspartic acid (μM)	0.10 ± 0.18	0.22 ± 0.21	0.00 ± 0.00	0.08 ± 0.13	0.21 ± 0.31
Asparagine (nM)	0.00 ± 0.00	0.00 ± 0.00	0.00 ± 0.00	0.00 ± 0.00	0.00 ± 0.00
Arginine (nM)	3041.50 ± 365.10	828.70 ± 619.70	2051.20 ± 406.20	1373.50 ± 548.70	2807.10 ± 2879.20
Cysteine (μM)	0.00 ± 0.00	0.00 ± 0.00	0.00 ± 0.00	0.00 ± 0.00	0.00 ± 0.00
Histidine (μM)	0.00 ± 0.00	0.00 ± 0.00	0.00 ± 0.00	0.00 ± 0.00	0.00 ± 0.00
Alanine (μM)	0.01 ± 0.01	0.00 ± 0.00	0.01 ± 0.01	0.01 ± 0.02	0.02 ± 0.03
Methionine (μM)	9.87 ± 4.46	6.41 ± 2.54	7.53 ± 4.64	14.17 ± 5.18	53.57 ± 66.53

[1]Values are presented as means ± SDs (n = 6).
[2]Values with the same letter in a column are not significantly different, as determined using a one-way analysis of variance followed by Duncan's multiple range test, $p < 0.05$.
[3]The C group was fed a control liquid diet for eight weeks; the E group was fed a control liquid diet for two weeks and then an ethanol-containing diet for six weeks; the AEGF-C group was fed an EGF-containing control liquid diet for eight weeks; the PEGF-E group was fed an EGF-containing control liquid diet for two weeks and then an ethanol-containing liquid diet for six weeks; and the AEGF-E group was fed an EGF-containing control liquid diet for two weeks and then an EGF-containing ethanol liquid diet for six weeks.

Supplementary Table S2. Effects of EGF on hepatic amino acid composition in rats fed with ethanol for 6 weeks[1,2].

Groups[3]	C		E		AEGF-C		PEGF-E		AEGF-E	
Valine (μM)	490.03 ± 214.99		424.14 ± 225.77		351.74 ± 177.48	0	392.44 ± 253.15		439.65 ± 223.30	
Leucine (μM)	632.26 ± 89.21		576.81 ± 198.43		559.64 ± 136.09		531.40 ± 177.04		531.07 ± 113.29	
Isoleucine (μM)	588.94 ± 164.20		477.11 ± 240.74		396.96 ± 142.53		479.96 ± 229.34		571.62 ± 333.51	
BCAA (μM)	1755.82 ± 350.20		1478.06 ± 59.28		1308.35 ± 325.96		1403.80 ± 588.77		1542.34 ± 655.31	
Tyrosine (μM)	443.92 ± 76.76		462.33 ± 83.51		363.80 ± 135.55		451.53 ± 92.47		392.98 ± 143.15	
Tryptophan (μM)	14.09 ± 5.70		14.55 ± 8.78		10.22 ± 3.66		13.69 ± 5.99		11.46 ± 4.27	
Phenylalanine (μM)	577.35 ± 28.78		537.32 ± 56.85		489.20 ± 116.38		495.32 ± 139.12		505.22 ± 143.80	
AAA (μM)	1035.37 ± 108.68		1014.21 ± 147.22		863.22 ± 254.66		960.54 ± 229.55		909.67 ± 290.76	
Threonine (μM)	58.31 ± 51.86		20.28 ± 5.98		15.95 ± 5.26		16.99 ± 4.71		21.45 ± 14.29	
Serine (nM)	0.00 ± 0.00		0.00 ± 0.00		0.00 ± 0.00		66.53 ± 115.24		0.00 ± 0.00	
Proline (μM)	116.97 ± 28.89		173.84 ± 119.83		184.45 ± 110.30		120.33 ± 63.23		164.06 ± 93.27	
Lysine (μM)	20.09 ± 3.93	b	38.13 ± 8.77	a	24.34 ± 2.44	ab	25.32 ± 14.76	ab	17.36 ± 7.28	b
Glycine (μM)	0.00 ± 0.00		0.00 ± 0.00		0.00 ± 0.00		0.00 ± 0.00		0.00 ± 0.00	
Glutamine (μM)	40.82 ± 3.57		40.90 ± 8.00		39.95 ± 5.95		33.74 ± 11.72		51.89 ± 14.79	
Glutamic acid (μM)	39.15 ± 17.68		35.61 ± 4.62		32.75 ± 2.83		37.20 ± 12.80		28.62 ± 4.94	
Aspartic acid (μM)	62.70 ± 38.29		53.72 ± 13.58		44.90 ± 40.94		57.51 ± 55.72		67.58 ± 58.55	
Asparagine (nM)	0.00 ± 0.00	b	0.00 ± 0.00	b	0.00 ± 0.00	b	41.67 ± 72.17	b	196.80 ± 171.99	a
Arginine (nM)	942.27 ± 247.77		984.87 ± 232.71		886.10 ± 433.91		754.37 ± 170.50		662.80 ± 191.48	
Cysteine (μM)	0.00 ± 0.00		0.00 ± 0.00		0.00 ± 0.00		0.00 ± 0.00		0.00 ± 0.00	
Histidine (μM)	26.48 ± 8.68	a	22.14 ± 19.17	ab	18.47 ± 10.81	ab	5.79 ± 5.80	ab	2.12 ± 0.69	b
Alanine (μM)	2.70 ± 0.70		3.82 ± 2.98		4.06 ± 1.45		2.49 ± 0.50		3.10 ± 1.39	
Methionine (μM)	80.64 ± 39.38		115.96 ± 129.33		101.09 ± 99.33		96.44 ± 111.17		97.64 ± 86.36	

[1]Values are presented as means ± SDs (n = 6).

[2]Values with the same letter in a column are not significantly different, as determined using a one-way analysis of variance followed by Duncan's multiple range test, $p < 0.05$.

[3]The C group was fed a control liquid diet for eight weeks; the E group was fed a control liquid diet for two weeks and then an ethanol-containing diet for six weeks; the AEGF-C group was fed an EGF-containing control liquid diet for eight weeks; the PEGF-E group was fed an EGF-containing control liquid diet for two weeks and then an ethanol-containing liquid diet for six weeks; and the AEGF-E group was fed an EGF-containing control liquid diet for two weeks and then an EGF-containing ethanol liquid diet for six weeks.

Supplementary Table S3. Effects of EGF on muscular amino acid composition in rats fed with ethanol for 6 weeks[1,2].

Groups[3]	C	E	AEGF-C	PEGF-E	AEGF-E
Valine (μM)	154.69 ± 4.11 ^{bc}	113.16 ± 48.67 ^{c}	231.77 ± 74.14 ^{ab}	201.33 ± 31.56 ^{a}	271.41 ± 79.05 ^{a}
Leucine (μM)	209.87 ± 83.99 ^{c}	377.11 ± 49.84 ^{b}	328.05 ± 50.97 ^{b}	353.59 ± 29.65 ^{b}	561.46 ± 66.38 ^{a}
Isoleucine (μM)	263.08 ± 65.46	234.22 ± 53.05	243.06 ± 91.01	249.04 ± 53.21	308.63 ± 37.90
BCAA (μM)	627.64 ± 127.74 ^{b}	724.49 ± 33.00 ^{b}	802.88 ± 109.99 ^{b}	803.96 ± 73.46 ^{b}	1141.50 ± 148.68 ^{a}
Tyrosine (μM)	268.32 ± 48.21	183.93 ± 74.07	225.18 ± 147.94	180.26 ± 58.40	296.17 ± 76.26
Tryptophan (μM)	9.25 ± 2.26 ^{ab}	7.00 ± 1.91 ^{b}	10.88 ± 0.93 ^{a}	10.87 ± 2.03 ^{a}	11.50 ± 1.97 ^{a}
Phenylalanine (μM)	246.68 ± 29.82	283.91 ± 78.33	325.84 ± 14.43	393.71 ± 136.77	352.07 ± 115.00
AAA (μM)	524.25 ± 45.16	474.85 ± 17.95	561.90 ± 159.53	584.84 ± 81.73	459.75 ± 70.23
Threonine (μM)	0.99 ± 1.72	1.66 ± 2.58	0.00 ± 0.00	0.00 ± 0.00	0.04 ± 0.07
Serine (nM)	0.00 ± 0.00	0.00 ± 0.00	0.00 ± 0.00	0.00 ± 0.00	0.00 ± 0.00
Proline (μM)	27.76 ± 4.90	26.49 ± 6.60	32.08 ± 10.27	21.14 ± 3.96	27.98 ± 8.18
Lysine (μM)	8.00 ± 1.29 ^{ab}	12.02 ± 5.00 ^{ab}	7.66 ± 1.40 ^{b}	6.41 ± 2.42 ^{b}	7.06 ± 1.77 ^{b}
Glycine (μM)	0.00 ± 0.00	0.00 ± 0.00	0.00 ± 0.00	0.00 ± 0.00	0.00 ± 0.00
Glutamine (μM)	16.77 ± 3.08 ^{a}	13.76 ± 3.81 ^{ab}	10.27 ± 0.66 ^{ab}	7.92 ± 1.01 ^{b}	14.34 ± 8.61 ^{ab}
Glutamic acid (μM)	24.20 ± 11.68	18.53 ± 5.31	16.01 ± 8.36	21.26 ± 11.50	25.99 ± 17.70
Aspartic acid (μM)	0.33 ± 0.12	3.90 ± 3.79	1.53 ± 1.39	1.78 ± 1.49	3.46 ± 3.31
Asparagine (nM)	93.87 ± 162.58	38.57 ± 48.34	30.30 ± 52.48	0.00 ± 0.00	0.00 ± 0.00
Arginine (nM)	1279.60 ± 304.52 ^{b}	3069.67 ± 629.23 ^{a}	1924.27 ± 150.23 ^{ab}	2114.97 ± 1099.07 ^{ab}	2600.50 ± 1176.68 ^{ab}
Cysteine (μM)	0.00 ± 0.00	0.00 ± 0.00	0.00 ± 0.00	0.00 ± 0.00	0.00 ± 0.00
Histidine (μM)	1.15 ± 0.37 ^{b}	11.53 ± 5.68 ^{a}	8.62 ± 7.67 ^{ab}	4.48 ± 5.97 ^{ab}	3.42 ± 3.90 ^{ab}
Alanine (μM)	1.72 ± 0.47	3.81 ± 3.73	4.38 ± 5.11	1.45 ± 0.39	1.64 ± 0.16
Methionine (μM)	28.78 ± 1.70 ^{bc}	22.77 ± 4.95 ^{c}	36.79 ± 9.10 ^{ab}	33.39 ± 3.49 ^{ab}	41.91 ± 2.03 ^{a}

[1]Values are presented as means ± SDs (n = 6).

[2]Values with the same letter in a column are not significantly different, as determined using a one-way analysis of variance followed by Duncan's multiple range test, $p < 0.05$.

[3]The C group was fed a control liquid diet for eight weeks; the E group was fed a control liquid diet for two weeks and then an ethanol-containing diet for six weeks; the AEGF-C group was fed an EGF-containing control liquid diet for eight weeks; the PEGF-E group was fed an EGF-containing control liquid diet for two weeks and then an ethanol-containing liquid diet for six weeks; and the AEGF-E group was fed an EGF-containing control liquid diet for two weeks and then an EGF-containing ethanol liquid diet for six weeks.

Supplementary Table S4. Experimental diet composition[1]

Ingredient[2]	Control	EGF[3]	Ethanol	Ethanol + EGF
	g/L (1000 kcal)			
Casein	41.4	41.4	41.4	41.4
L-Cysteine	0.5	0.5	0.5	0.5
DL-Methionine	0.3	0.3	0.3	0.3
Corn Oil	8.5	8.5	8.5	8.5
Olive Oil	28.4	28.4	28.4	28.4
Safflower Oil	2.7	2.7	2.7	2.7
Choline bitartrate	0.53	0.53	0.53	0.53
Fiber	10	10	10	10
Xanthan gum	3	3	3	3
AIN-76 Vitamins	2.5	2.5	2.5	2.5
AIN-76 Minerals	2.6	2.6	2.6	2.6
Maltodextrin	115.2	115.2	25.6	25.6
Ethanol	-	-	50	50
EGF[3] (μg/kg BW/day)	-	30	-	30

[1]The C group was fed a control liquid diet for eight weeks; the E group was fed a control liquid diet for two weeks and then an ethanol-containing diet for six weeks; the AEGF-C group was fed an EGF-containing control liquid diet for eight weeks; the PEGF-E group was fed an EGF-containing control liquid diet for two weeks and then an ethanol liquid diet for six weeks; and the AEGF-E group was fed an EGF-containing control liquid diet for two weeks and then an EGF-containing ethanol liquid diet for six weeks. [2]Casein, AIN-76 vitamins, AIN-76 minerals, L-cysteine, DL-methionine, choline bitartrate, fiber, and maltodextrin were purchased from MP Biomedicals (Irvine, CA, USA). Ethanol was obtained from Sigma-Aldrich (St. Louis, MO, USA). Corn oil was obtained from God Bene Enterprise (Yunlin, Taiwan). Olive oil was obtained from Standard Foods Corporation (Taipei, Taiwan). Safflower oil was purchased from Taiwan Sugar Corporation (Taipei, Taiwan). [3]EGF was purchased from PeproTech (Cranbury, NJ, USA).

Supplementary Table S5. Antibodies for analyzing related proteins.

Sample	Antibody	Source
Liver	Mouse monoclonal TLR4	Santa Cruz Biotechnology, Dallas, TX, USA
	Mouse monoclonal MyD88	Proteintech Group, Rosemont, IL, USA
	Rabbit polyclonal TRIF	Abcam, Cambridge, UK
Intestine	Rabbit polyclonal Claudin-1	Affinity Bioscience, Cincinnati, OH, USA
	Rabbit polyclonal Occludin	Affinity Bioscience, Cincinnati, OH, USA
Muscle	Rabbit polyclonal 70S6K	Affinity Bioscience, Cincinnati, OH, USA
	Rabbit polyclonal p-70S6K-T389	ABclonal Technology, Woburn, MA, USA
	Rabbit polyclonal Myostatin	Proteintech Group, Rosemont, IL, USA
Internal control	Mouse monoclonal GAPDH	Millipore, Burlington, MA, USA
	Mouse monoclonal β-actin	Santa Cruz Biotechnology, Dallas, TX, USA

TLR4, Toll-like receptor 4; Myd88: myeloid differentiation primary response 88, TRIF: TIR-domain-containing adapter-inducing interferon-β; 70S6K, 70 ribosomal protein S6 kinase; GAPDH, glyceraldehyde 3-phosphate dehydrogenase.

Supplementary Table S6. List of primer sequences for analyzing mRNA.

	Forward 5′→3′	Reverse 5′→3′
FOXO	TCAAGGATAAGGGCGACAGC	GTTCCTTCATTCTGCACTCGAAT
MURF	TAGAAGCTTCCAAGGGCTGC	GTGTCCCTCTGTGGACACG
Atrogin	CTTCTCGACTGCCATCCTGG	CTCTGGACCAGCGTGCATAA
GAPDH	AGTGCCAGCCTCGTCTCATA	GATGGTGATGGGTTTCCCGT

Akt, protein kinase B; mTOR, mammalian target of rapamycin; LC3B, microtubule-associated proteins 1A/1B light chain 3B; FOXO, forkhead box protein O1; MuRF-1, muscle ring-finger protein-1; S6K, ribosomal protein S6 kinase; 4EBP1, eukaryotic translation initiation factor 4E (eIF4E)-binding protein 1; GAPDH, glyceraldehyde 3-phosphate dehydrogenase.

Supplementary Table S7. Chemicals and kits used in this study.

Items	Chemical/Kit	Source	Catalog/CAS no.
Diet-related products	Casein	MP Biomedical, Santa Ana, CA, USA	0290129325
	L-Cystine	MP Biomedical, Santa Ana, CA, USA	0210145490
	DL-Methionine	MP Biomedical, Santa Ana, CA, USA	190955 / 59-51-8
	Choline Bitartrate	MP Biomedical, Santa Ana, CA, USA	0210138490
	Alphacel non-nutritive (Fiber)	MP Biomedical, Santa Ana, CA, USA	0290045325
	Xanthan gum	CP Kelco US Inc., Okmulgee, OK, USA	KELTROL 521-XANTHAN GUN
	ICN: AIN-76 Vitamins	MP Biomedical, Santa Ana, CA, USA	0290545401
	ICN: AIN-76 Minerals	MP Biomedical, Santa Ana, CA, USA	0290545502
	Dextrinazed corn starch	MP Biomedical, Santa Ana, CA, USA	0296042925
	Ethanol (99.8%)	Honeywell Research Chemicals, Charlotte, NC, USA	32221-2.5L / 64-17-5
Chemicals used in this study	NaCl	Honeywell Research Chemicals, Charlotte, NC, USA	31434S / 7647-14-5
	Tris–HCl	J. T. Baker, Radnor, PA, USA	4103-01 / 1185-53-1
	Sodium dodecylsulfate (SDS)	Sigma-Aldrich, St. Louis, MO, USA	L5750 / 151-21-3
	Nonyl phenoxypolyethoxylethanol (NP-40)	Cayman Chemical, MI, USA	700024
	Chloroform	Riedel-de Haën, Honeywell Research Chemicals, Charlotte, NC, USA	32211 / 67-66-3
	Isopropanol	J. T. Baker, Radnor, PA, USA	9084-01 / 67-63-0
	Polyethylene glycol mono-p-isooctylphenyl ether (Triton X-100)	Nacalai tesque, INC., Kyoto, Japan	282-29 / 9002-93-1
	Tris-Base	Bioman scientific Co., LTD., New Taipei City, Taiwan	TRS011.1
	Glycine	Bioman scientific Co., LTD., New Taipei City, Taiwan	GLN011.1
	30% Acrylamide	Bio-rad, Hercules, CA, USA	1610158
	TEMED	Sigma-Aldrich, St. Louis, MO, USA	T7024 / 110-18-9
	Ammonium persulfate	Sigma-Aldrich, St. Louis, MO, USA	A3678 / 7727-54-0
	Methanol	ECHO Chemical, Miaoli County, Taiwan	MA1101
	Tween 20	Sigma-Aldrich, St. Louis, MO, USA	P-7949 / 9005-64-5
	10% Formaldehyde solution	Macron fine chemicals, Radnor, PA, USA	H121-08
	Acetonitrile	J. T. Baker, Radnor, PA, USA	9017-03 / 75-05-8
	Formic acid	Nacalai tesque, INC., Kyoto, Japan	163-11 / 64-18-6
	TRI Reagent®	Sigma-Aldrich, St. Louis, MO, USA	T9424
	2X RIPA Buffer	BioBasic INC., Amherst, NY, USA	RB4475

	Proteinase inhibitor cocktail	MedChemExpress, Monmouth Junction, NJ, USA	HY-K0010
	Phosphatase inhibitor cocktail tablets	Roche, Basel, Switzerland	4906837001
ELISA kits	IL-1β	R&D Systems, Minneapolis, MN, USA	dy501
	IL-6	R&D Systems, Minneapolis, MN, USA	dy506
	IL-10	R&D Systems, Minneapolis, MN, USA	dy522
	TNF-α	R&D Systems, Minneapolis, MN, USA	dy510
Colorimetric assay kits	Thiobarbituric acid reactive substance (TBARS)	Cayman Chemical, MI, USA	10009055
	Triglyceride	Cayman Chemical, MI, USA	10010303
	Cholesterol	Cell Biolabs, San Diego, CA, USA	STA-384
PCR related kits	RevertAid First Strand cDNA Synthesis kit	ThermoFisher Scientific, Waltham, MA, USA	K1621
	SYBR Green/ROX qPCR Master Mix	ThermoFisher Scientific, Waltham, MA, USA	K0222
Western blot-related kit	4X SDS Loading Dye	Bioman Scientific Co., LTD., New Taipei City, Taiwan	P1001
	Prestained Protein Ladder	Bioman Scientific Co., LTD., New Taipei City, Taiwan	Prep1025
	Prestained Protein Ladder	Bioman scientific Co., LTD., New Taipei City, Taiwan	Prep1025G
	T-Pro LumiLong Plus Chemiluminescence Detection Kit	T-Pro Biotechnology, New Taipei City, Taiwan	JT96-K004M

Sample	Antibody	Source	Catalog
Liver	Mouse monoclonal TLR4	Santa Cruz Biotechnology, Dallas, TX, USA	A0620
	Mouse monoclonal MyD88	Proteintech Group, Rosemont, IL, USA	66660-1-1g
	Rabbit polyclonal TRIF	Abcam, Cambridge, UK	Ab13810
Intestine	Rabbit Polyclonal Claudin-1	Affinity Bioscience, Cincinnati, OH, USA	AF0127
	Rabbit Polyclonal Occludin	Affinity Bioscience, Cincinnati, OH, USA	DF7504
Muscle	Rabbit polyclonal Myostatin	ABclonal Technology, Woburn, MA, USA	A6913
	Rabbit polyclonal S6K	Affinity Bioscience, Cincinnati, OH, USA	AF6226
	Rabbit polyclonal p-S6K-T389	ABclonal Technology, Woburn, MA, USA	AP0564
	Rabbit monoclonal p62	Cell Signaling, Danvers , MA, USA	5114
	Rabbit monoclonal Beclin1	Genetex, Irvine, CA, USA	GTX133555
	Rabbit monoclonal MuRF-1	Proteintech Group, Rosemont, IL, USA	55456-1-AP
	Rabbit polyclonal SMAD2/3	ABclonal Technology, Woburn, MA, USA	A7536
Internal control	Mouse monoclonal GAPDH	Proteintech Group, Rosemont, IL, USA	HRP-60004
	Mouse monoclonal β-actin	Proteintech Group, Rosemont, IL, USA	66009-1-Ig

Secondary antibody	Goat anti-Rabbit IgG (H+L) HRP	Croyez. Taipei, Taiwan	C04003
	Peroxidase-conjugated Affinipure Goat anti-Mouse IgG (H+L)	Jackson ImmunoResearch INC., West Grove, PA, USA	115-035-033
